# A Focusing Method in the Calibration Process of Image Sensors Based on IOFBs

**DOI:** 10.3390/s100100047

**Published:** 2009-12-24

**Authors:** Pedro R. Fernández, José L. Lázaro, Alfredo Gardel, Ángel E. Cano, Ignacio Bravo

**Affiliations:** 1 Electronics Department, Superior Polytechnic School, University of Alcalá, Universitary Campus, Alcalá de Henares 28871, Madrid, Spain; E-Mails: pedro.fernandez@depeca.uah.es (P.R.F.); alfredo@depeca.uah.es (A.G.); ibravo@depeca.uah.es (I.B.); 2 Telecommunication Department, University of Oriente, Av. de las Américas, SN, Santiago de Cuba 90900, Cuba; E-Mail: angel.cano@depeca.uah.es

**Keywords:** image sensor, image transmission, sensor calibration, optical fiber sensors

## Abstract

A focusing procedure in the calibration process of image sensors based on Incoherent Optical Fiber Bundles (IOFBs) is described using the information extracted from fibers. These procedures differ from any other currently known focusing method due to the non spatial in-out correspondence between fibers, which produces a natural codification of the image to transmit. Focus measuring is essential prior to carrying out calibration in order to guarantee accurate processing and decoding. Four algorithms have been developed to estimate the focus measure; two methods based on mean grey level, and the other two based on variance. In this paper, a few simple focus measures are defined and compared. Some experimental results referred to the focus measure and the accuracy of the developed methods are discussed in order to demonstrate its effectiveness.

## 1. Introduction

IOFBs can be used for image transmission over longer distances and at lower cost than conventional coherent fiber bundles. In all cases, an information “decoding” or “calibration” process has been demonstrated to be necessary because of the non-spatial correspondence between the input and output fiber spatial distribution [1]. Various strategies for this exist, but in general, the calibration process is carried out by scanning the input under controlled settings, and verifying the output response sensed by an area scan camera. Results are recorded on a LUT (Look-up table) in the memory and the decoding rule is obtained using this information. The Authors’ solution can be consulted in [1,2].

**Figure 1.**
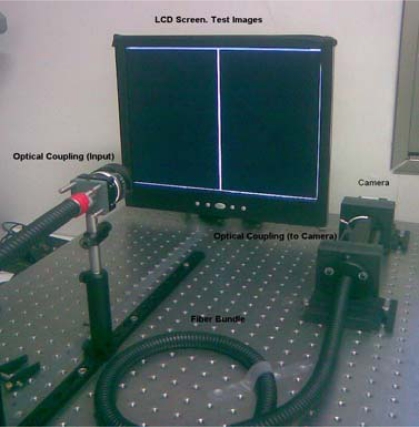
System setup.

Previous research has described a fiber location method using morphological processing and distance transform “*Fiber Detection, using Distance Transform”* (FDDT), together with a calibration method [1]. The former is extremely important for particularizing fiber behaviour and location in front of the camera, using a setup as shown in Figure 1. This comprises:

- An LCD monitor, which acts as a light source in FDDT, illuminating the IOFB homogenously. This element is used in calibration to project a serial image of known patterns onto the IOFB entrance.
- A PC or similar unit to control and process the images emitted by the sensor.
- A CMOS camera, which acts as a sensor and is controlled by the PC, for capturing images of the IOFB output face.
- Several additional accessories, including the lens couplers.
- An IOFB, which acts as a transport element.

The location of the fibers is defined during a pre-calibration task known as Fiber Detection, using Distance Transform (FDDT). Some advantages of this process are outlined below:

- Location of fibers on the outside face of an IOFB. These locations are the first elements included on the Lookup Table (LUT) as output parameters, where the captured input information is redistributed.
- A reduced number of coordinates to process in bundle calibration and decoding process is required.
- Fiber response can be particularized if each one is stimulated homogenously.

In [2], a calibration technique, comprising line scanning at the entrance of the bundle, is described. This method produces good image reconstruction, reliable results and is less time-consuming than other techniques. However, in spite of recognizing the necessity for focusing prior to calibration, no detailed implementation techniques are provided.

Notice that analyzing [2] we could think that, in fact, we could first generate the LUT for image reconstruction and later focus this image. However, if the process starts with system calibration it produces an energy spreading defocus- on the bundle input and therefore LUT generation could be erroneous.

In this paper, a model for a system comprising an IOFB and an input optic is analyzed, and the concept of focusing is described. This latter is necessary in order to understand the methodology which follows. Lastly, the qualitative results obtained from the focusing measures and the equalization methods described in the preceding sections are discussed.

## 2. Background

**Figure 2.**
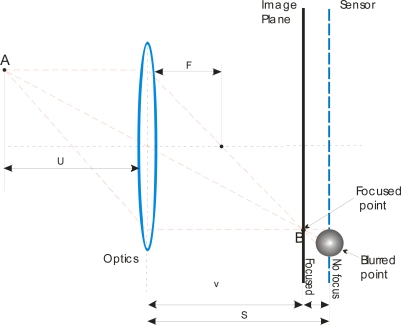
Image formation and focusing.

A focus measure is a value which describes the degree of blurring of an image formed on a sensor. This magnitude should be a global maximum when the image is sharply focused, decreasing as blurring increases [3]. Figure 2 shows a basic image formation scheme. All rays radiate from *A*, passing through the lens to converge on point *B* on the image plane. Nevertheless, on the sensor plane the point appears in a blurred region with a determinate diameter. The focused lens position *v* depends on the distance *u* of the object to be focused and the focal distance *f* of the lens. This can be expressed as:

(1) $$\frac{1}{f}=\frac{1}{u}+\frac{1}{v}$$

When point *A* is blurred on the CMOS sensor, it is imaged as a blurred circle of determinate radius. This blurred image *h*(*i,j*) constitutes a Point Spread Function (PSF). In general, the blurring system is modeled as a linear space-invariant imaging system as follows [4]:

(2) g(i,j)=h(i,j)*f(i,j)+n(i,j)

The blurred image *g*(*i,j*) is equal to the convolution of the original image *f*(*i,j*) and the PSF *h*(*i,j*) of the blurring system. Additional noise *n*(*i,j*) is assumed with zero-mean. In most cases, the out-of-focus blur in a circular aperture system can be modeled as a uniform disk of radius *R*, ignoring diffraction:

(3) $$h(x,y)=\left\{\begin{array}{l} 0\overset{}{\to }\sqrt{x^{2}+y^{2}}>R\\ \frac{1}{\pi R^{2}}\to \sqrt{x^{2}+y^{2}}\leq R \end{array}\right\}$$

In our case, we have a completely messy image and can’t focus it, because it is not recognizable. But it is necessary to focus it in order to generate the LUT. If we analyze [2] we can notice that focusing process must be prior to LUT generation in order to avoid erroneous correspondences between input and output points, and possible effect of redundancies in LUT registers. Furthermore, an inappropriate focus could produce energy dispersal, which would affect estimation of fiber response as the energy which impacted on a fiber would be less than that which it should receive during focusing. The system would be poorly calibrated, and reorganization of information of interest would be impaired.

**Figure 3.**
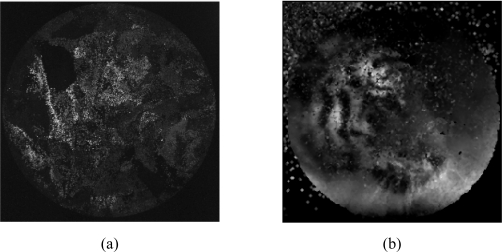
Reconstruction result using a process beginning with a calibration procedure. (a) Original image captured by the sensor. (b) Reconstructed image

Figure 3 shows the result for the reconstruction process. Figure 3a presents the original image generated by the output side of bundle and captured by the sensor. Figure 3b shows the result for a process that firstly calibrates and later focuses.

## 3. Proposed Focusing Measures Process

Therefore, ignoring the noise element and considering that *G*, *H* and *F* are the Fourier transform of *g*, *h*, and *f* respectively, then *G = HF*. The OTF (Optical Transfer Function) *H*(*w,v*) corresponding to *h*(*x,y*) is circularly symmetric, and its cross section seems to be a *sinc* function [5] in which the first zero depends on the degree of focus. This behavior constitutes a low pass filter. Therefore, for a focused image, the first zero is further from the origin in the spectrum, and for a non-focused image, the higher frequencies are reduced, thus increasing the blurring effect.

For an operator, is necessary to use a parameter or measure which describes the focusing level when the optics are changed or adjusted. The focus measure comes closest to maximum with the best focused image, and decreases as the image blurs.

The focus measurement should accomplish the following [6]:

- Content-independent: it should not be influenced by any particular structure in the image, such as brighter points, *etc*.
- Monoticity: it should decrease monotonically above and below the focus position
- Good power of discrimination and accuracy: it should give a sharper response when the focus point is closer. The sharper the focus, the easier it is to focus the system accurately. The focus value should be able to combat the effect of noise and low-contrast imaging conditions.
- Applicability: it should work well for any reasonable sample and conditions.
- Implementation: it should be easy to implement and efficient.

It should be noted that that in the reconstruction of the image, only those points detected by FDDT are used (due to the increase in quality and the significant reduction in time taken to reconstruct the image that this step contributes). Thus, given that FDDT will be implemented and used for reconstruction, it is also used in order to optimize the focus specifically on those particular points which will determine the future image.

We would like to specify that the points close to the fiber perimeter are contaminated by the influence of energy from other adjoining fibers when the image is formed in the receiver. Consequently, the use of points close to the fiber center for both focusing and reconstruction, and thus the use of FDDT as a means of locating them, is fully justified.

Time is proportional to the number of points (without FDDT, 6.5 M points, and with FDDT, approximately 50 K points). Quality is reflected in the fact that image noise (interstices, broken fibers, variable fiber attenuation, *etc*.) is eliminated, since only the gray values in the centre of each fiber are used.

### 3.1. Focusing Model for an Incoherent Fiber Bundle

Focus Measuring in IOFBs for imaging is an important task due to the natural codification of the image transmitted by the IOFB. In these systems, the IOFB output side is always focused on the sensor; nevertheless this is not necessarily true for the input side of the bundle. Images on the output side do not have direct information concerning the edges of the real image, or the gradient, color changes, frequency spectrum, *etc*., all of which comprise important data in many focus measures described in the literature [3–10]. These methods take into account the progressive behaviour of the image parameters depending on the optic position. Nevertheless, in images transmitted by IOFB, it is impossible to determine the focus measure using the above mentioned parameters because of the disorder or codification of the image. Consequently, the necessary information is lacking until the system has been calibrated.

The question is, how to verify that the input optics are focused on the scene correctly, and how to be sure that the calibration process has resulted in accurate focusing of the input optics, when the image status on the output side is still unknown? Consequently, a focus procedure is needed in order to ensure correct calibration.

Firstly, it will be necessary to describe a focusing model for a fiber bundle coupled by standard input optics. The system’s measure element is the CMOS sensor, which measures the focus from the disordered image transmitted through the IOFB. This information can only be extracted from the core of the fiber locations.

**Figure 4.**
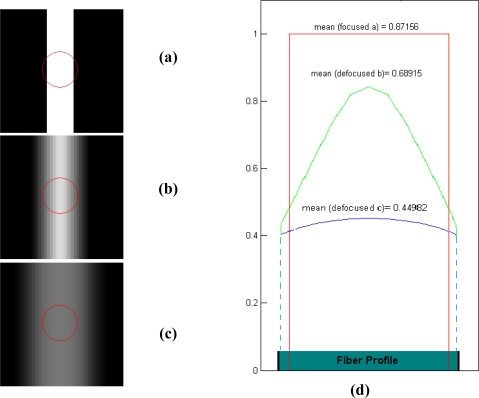
Simulation of the fringe superposition on fibers (circle) when focus is changing. (a) focused case. (b) and (c) defocused cases. (d) irradiance profile on the fiber.

Figure 4 shows a simulation where a strip of light is projected onto a fiber, as happens when a line coming from the test bank’s LCD overlaps a fiber. Three different focus degrees are represented. The energy distribution on the fiber face depends on the focus. The fiber response depends both on the superposition area between the fringe and the fiber face, and the focus position on the optics. In Figure 4a, a focused strip has a sharper border and a strong influence on the fiber. In this case, the highest response will be achieved (Figure 4d). Nevertheless, if a PSF is used to model the blurring effect of the optics in an unfocused position, the response of the fiber will fade. This is because the irradiance received is affected by the PSF.

The irradiance *E*(*x,y*) on the fiber can be modeled as *0* on the outside of the fiber core with radius *r_f_*, whilst that absorbed by the fiber depends on the region *R* where the light is projected with optical power *P_E_*. The optical power collected by the fiber is obtained by assessing the overlapping area of a circle and a rectangle provided by the projected strip and generated by the monitor.

(4) $$E(x,y)=\left\{\begin{array}{l} 0\to \sqrt{x^{2}+y^{2}}>r_{f}\\ \frac{P_{E}}{\pi r_{f}{}^{2}}R\to \sqrt{x^{2}+y^{2}}\leq r_{f} \end{array}\right\}$$

On the output side of the fiber, the maximum grey level *gl_max_* (at the fiber center) sensed by the camera is proportional to the mean value of *I* and consequently depends on the focus:

(5) $${gl}_{\text{max}}\equiv E(x,y)$$

Taking into account the simulation presented in Figure 4, it is possible to establish that a direct means of obtaining a focus measure is to evaluate the grey levels obtained from the centroids of a group of fibers and captured by the sensor, stimulated by the scanning line.

Polling a group of fibers is an attractive idea because the superposition cases, shown in the above simulation, are ideal. In an IOFB, it is impossible to guarantee that the line or a spot will overlap at the center of the illuminated fibers. Therefore, the grey levels sensed by the camera decrease in some cases, whilst in others the opposite occurs. In order to avoid this, the best illuminated regions can be selected by a thresholding process, where those which are best illuminated represent the most realistic cases. Thus, the *gl_mean_* value can be calculated from the means of all the *gl_max_* values for those *K* coordinates which do not reach the threshold value on the image, without taking into account the background (noise).

(6) $${gl}_{\mathrm{mean}}=\frac{1}{K}\;\sum_{i=1}^{K}{gl}_{\text{max}}\;(i)$$

Another accurate focusing measure consists in calculating the energy from the total image [5,8], yielding a maximum value in the focused position.

Using FDDT, the grey levels obtained from the fiber cores can be estimated easily, and the quantity of data needed to process (*K*) can be reduced notably. The *K* centroid coordinates on the Image *I(u,v)* obtained from FDDT, enables the measure to be calculated thus:

(7) $${gl}_{\mathrm{mean}}=\frac{1}{{Kk}^{2}}\sum_{i=1}^{K}\sum_{x=u(i)-\frac{\left(k-1\right)}{2}}^{u(i)+\frac{\left(k-1\right)}{2}}\sum_{y=v(i)-\frac{\left(k-1\right)}{2}}^{v(i)+\frac{\left(k-1\right)}{2}}I(x,y)\;\;\;\;\;k\geq 1\;\;\text{odd}\;\text{values}$$

The grey level parameter can be obtained by using a mean value of the pixels near the center of the fiber (*kxk* square region).

Variance of image intensity can be used as a sharpness measure. Variance has been used in standard focusing procedure and its robustness to noise has been previously noted in [5,6,8].

Variance should peak with the best focus, since in-focus images will have greater intensity variation than blurred, defocused images. In the case of IOFB, information is extracted from the fiber cores represented on the CMOS sensor (applying FDDT method) and the measure can be written as:

(8) $$f_{\text{var}}=\frac{1}{K}\sum_{i=1}^{K}{\left[\mathrm{gl}(i)-{gl}_{\mathrm{mean}}\right]}^{2}\;\;\;\;\;\;\;\;\;\text{gl}\geq {\text{gl}}_{\text{thresh}}$$

where *gl* is the mean grey level using a *kxk* window centered on each fiber core, and *gl_mean_* is the mean grey level extracted from all the *K* fiber cores.

Because of the low variance in the energy in the fiber cores, the above equation can be rewritten for an *MxN* image as:

(9) $$f_{\text{var}}=\frac{1}{\mathrm{MN}}\sum_{x=1}^{N}\sum_{y=1}^{M}{\left[I(x,y)-I_{\mathrm{mean}}\right]}^{2}$$

where *I*(*x,y*) is the grey level in the (*x,y*) pixel position and *I_mean_* is the mean grey level in the image. In both cases, the measures are valid but in (9), an FDDT procedure is unnecessary because the whole of the image is analyzed. However, the influence of image noise, especially on background, is taken into account.

Equations in (6–9) represent four alternatives for focus measures, which can be used in order to determinate the best focus accuracy. In order to decide which of the measures analyzed are the most effective, and in order to conduct comparisons with other methods proposed by other researchers, the standard deviation of focus measures around the focus point *f_0_* can be used. In [5,8], Subbarao introduces a metric named Auto-focusing Uncertainty Measure (AUM) which is useful in selecting the most accurate focus measure from a given set of focus measures.

Let *F*, a function which correlates the focus measure with lens position, if *F* is known, the standard deviation σ can be obtained, by means of which the factor *Q = f_2_−f_1_* can be calculated, where *f_1_ < f_0_ < f_2_* are the lens positions which correspond to *|F(f_0_)-F(f_1_)|=|F(f_0_)-F(f_2_)|=*σ. *Q* provides information concerning the power of discrimination and the sharpness of *F* in the proximity of *f_0_*. The lower the value for Q, the more unique the optimal focus value.

## 4. Focusing Results

Tests were done using a Pentium IV HT 3 GHz CPU with 2 Gigabytes of RAM. The camera was a monochromatic CMOS camera BCi-6600 (6.6 Megapixels) that was empirically coupled with a flexible optic-fibre light guide with approximately 50,000 fibres 50 μm in diameter. This camera granted sufficient resolution (about 7 × 7 pixels per fibre) for the detection process and image reconstruction. Algorithms were developed and compared with Matlab 7.1.

The focusing procedure can be implemented using the setup described in the “background” section of this paper. An image with highly contrasting details can be useful for identifying the correct position in the optic subsystem. This can be achieved using a bright fringe with a width sufficient for the area of impact to reach almost 3/4s of fiber diameter.

**Figure 5.**
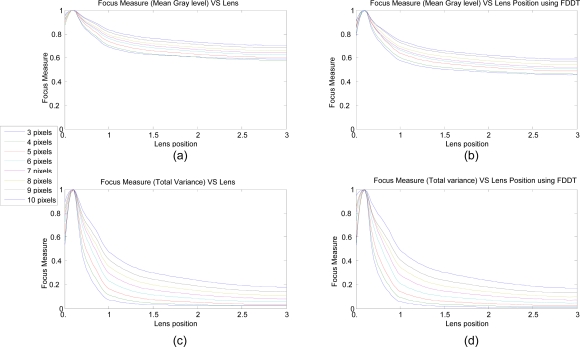
Focus measures for different optic focus position and methods. (a) *gl_mean_* and (b) *f_var_* without previous fiber detection. (c) *gl_mean_* and (d) *f_var_* using previous fiber detection.

A line under a black background provides clear differentiation for both measures, because the energy is easily scattered and thus varies the focus. To capture an image sequence for each optic position, the measure parameter is determined using the same experimental conditions and camera parameters. Figure 5 shows the normalized behavior for different fringe widths, using the four different focus measures described previously. Each color corresponds to a fringe width.

As can be seen in Figure 5, the local maximums are always reached in the same position and the response is in accordance with rules for focus measure, especially as regards monotone, discrimination power, and accuracy. This is more appropriate, especially when the focus adjustment of the optics is logarithmic. The graphics were obtained using 19–35 mm zoom optics from Cosina.

The measures were performed under an iterative loop which included a capturing procedure and a measure calculus. The time required depends not only on the capture time and processing of the images, but also on the number of samples sensed per image. It is not necessary to include all of the points derived from the core of the fibers or the image in the analysis, thus reducing the time needed to acquire the measure without loss of information.

The procedure described here is only necessary when the system is not calibrated. Once calibration has been carried out, focusing can be performed manually for a fixed working distance.

**Figure 6.**
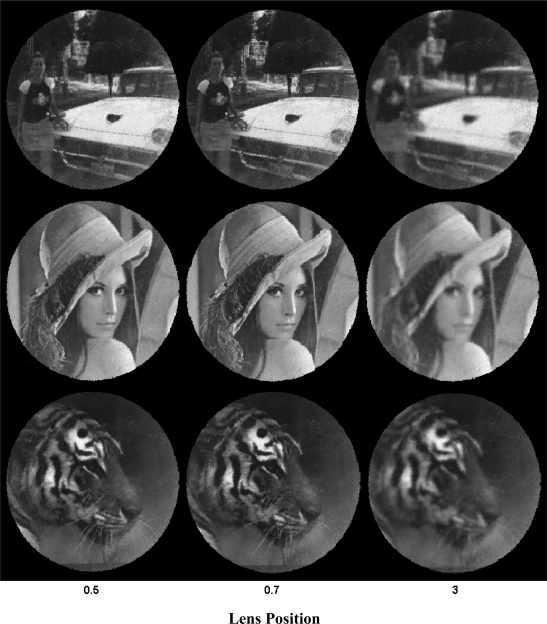
Real Image reconstruction using an 19–25 mm optics in different focus positions. The central images (0.7) correspond with the best focus position.

A simple demonstration can be carried out using a calibrated system in order to demonstrate the coherence of the measures. This reverse procedure consists in capturing a fixed image from the screen using different optic positions in order to obtain the best focus results. For each image the focus is verified. Figure 6 shows a reconstructed sequence of different images using 25 mm optics from Electrophysics with a relative aperture of 1:1.4

In order to quantify the best focus, a correlation parameter is calculated between reference image and reconstructed images. The following table shows the results for each optic position. The values in black represent the best result which coincides with the best focus (Figure 6).

**Figure 7.**
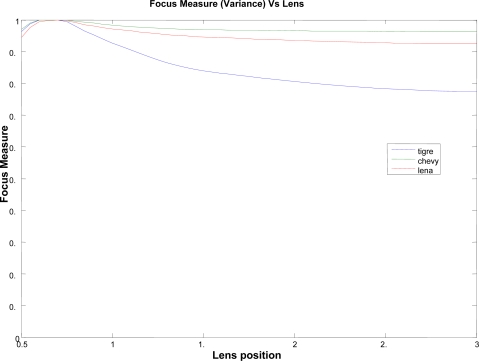
Focus measure for different images and lens position.

Notice that this analysis only can be done by using high contrast images. Images, as shown in Figure 5, have poor response and discrimination power. Figure 7 shows the results for the above represented samples, obtained from applying the variance algorithm [using Equation (9)]. A peak can be seen near the position 0.7 in all of the images, denoting the correct focus position. Nevertheless, a response dependence on image features is also visible, especially for the variance measure.

**Figure 8.**
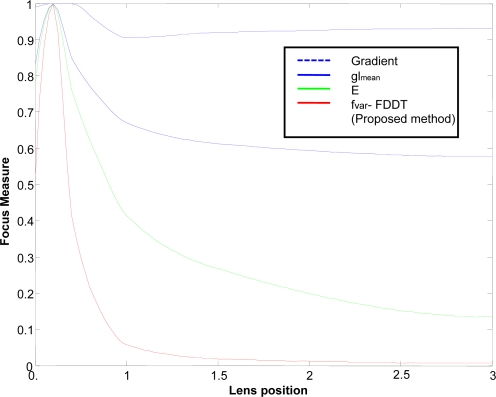
Focus measure for different images and lens position.

In order to verify our results, we analyzed the different focus functions F *vs.* the lens position (Figure 8), using a fringe width of 3 pixels on a black background as the reference image. The variance measure and the mean of image intensity levels were compared with other methods based on gradient and gray level, defined in [5,8], and on measures of energy E of the image as suggested in [9].

Note that methods based on FFT and the Laplacian consider information from the fiber edges rather than the useful information obtained from the transmitted image (edges, intensity changes, *etc.*). Therefore, they were not included in this comparison as their results would be poor and unclear in this application.

Table 2 shows comparative results for each analyzed measure applying Q metric, introduced by Subbarao [5]. Thus, these results should be treated primarily as a relative measure of performance.

In the light of the results obtained (Table 2), we can conclude that the best focus measure which complies with our working conditions is the measure of variance using FDDT (a method developed and proposed by the authors). The need to detect fibers does not represent a difficulty, given that this information is necessary for the subsequent calibration process, and the FDDT algorithm is rapid and accurate.

## 5. Conclusions

Image transmission by means of IOFB is possible, but it is not exempt from certain technical difficulties rooted in the ineludible task of calibration, permitting the final reconstruction of any image projected on the entrance of the bundle. One of the main problems faced in before calibration consists in determining the best focus position in order to grant a good image reconstruction.

In this paper, four focus measures, basically using the grey levels on the sensor and the variance, are defined. This focus procedure is carried out prior to calibration in order to ensure accurate performance. The principle disadvantage of these measures is their dependence on the characteristics of the image, which can affect the precision of the measure. However, this problem can be minimized by using fringes or spot formed test images. Once the focus position has been obtained, calibration can be carried out with a high probability of success.

The variance measure presents a better discrimination power and a higher range of possible values being a more interesting magnitude for focus determination. These methods of measurement were implemented on the setup PC and were verified under real situations obtaining good focus determination. Once the calibration is completed, the methodology described here is not necessary, because the transmitted image can be now reconstructed directly.

Four algorithms were developed to estimate the focus measure. Two variants use the average value of gray level or variance of the image, taking into account the values exceeding a certain threshold. The other two methods calculate the focus using information obtained from the centers of the fibers using FDDT. Thus, a smaller number of coordinates are processed so that the computing time is reduced.

## Figures and Tables

**Table 1.** Correlation results.

**File**	**Chevrolet**	**Lena**	**Tiger**
**Lens Posit.**			
0.5	0.87809	0.90179	0.79068
0.6	0.89811	0.9265	0.84814
**0.7**	**0.92416**	**0.94232**	**0.86918**
0.85	0.91523	0.93729	0.85346
1.0	0.90773	0.93455	0.838
1.5	0.89771	0.93013	0.81185
3.0	0.89549	0.92973	0.80978

**Table 2.** Comparative results.

**Measure**	***Q***
Gradient	0.2750
gl_mean_	0.1850
Image energy *E*	0.1500
**f_var_using FDDT (proposed method)**	**0.0750**
